# Hepatocyte‐derived exosomes deliver the lncRNA CYTOR to hepatic stellate cells and promote liver fibrosis

**DOI:** 10.1111/jcmm.18234

**Published:** 2024-03-23

**Authors:** Wenqiang Xu, Wenhui Mo, Dengyu Han, Weiqi Dai, Xiaorong Xu, Jingjing Li, Xuanfu Xu

**Affiliations:** ^1^ Department of Gastroenterology Shidong Hospital of Shanghai, School of Health Science and Engineering, University of Shanghai for Science and Technology Shanghai China; ^2^ Department of Gastroenterology Shanghai Tenth People's Hospital, Tongji University School of Medicine Shanghai China

**Keywords:** ceRNA, exosomes, hepatic stellate cells, liver fibrosis, lncRNA‐CYTOR

## Abstract

Liver fibrosis is characterized by the activation and transformation of hepatic stellate cells (HSCs) induced by various injury factors. The degree of liver fibrosis can be significantly improved, but persistent injury factors present a significant therapeutic challenge. Hepatocytes are the most important parenchymal cell type in the liver. In this study, we explored the molecular mechanisms by which damaged liver cells activate HSCs through extracellular vesicles. We established a coculture model of LO2 and LX2 and validated its exosomal transmission activity. Subsequently, differentially expressed long noncoding RNAs (lncRNAs) were screened through RNA sequencing and their mechanisms of action as competing endogenous RNAs (ceRNAs) further confirmed using biological methods, such as FISH and luciferase assays. Damaged liver cells induced activation of LX2 and upregulation of liver fibrosis‐related markers. Exosomes extracted and identified from the supernatant fraction contained differentially expressed lncRNA cytoskeleton regulator RNA (CYTOR) that competed with microRNA‐125 (miR‐125) for binding to glial cell line‐derived neurotrophic factor (GDNF) in HSCs, in turn, promoting LX2 activation. MiR‐125 could target and regulate both CYTOR and GDNF and vice versa, as verified using the luciferase assay. In an in vivo model, damaged liver extracellular vesicles induced the formation of liver fibrosis. Notably, downregulation of CYTOR within extracellular vesicles effectively inhibited liver fibrosis. The lncRNA CYTOR in exosomes of damaged liver cells is upregulated and modulates the expression of downstream GDNF through activity as a ceRNA, providing an effective mechanism for activation of HSCs.

## 1 INTRODUCTION

Liver fibrosis is a key link in the progression of various chronic liver disorders, from viral hepatitis and fatty liver to end‐stage liver disease. One common molecular mechanism is the activation of hepatic stellate cells (HSCs) by various injury factors and their transformation into myofibroblasts (MFs).^1^, ^2^ Earlier research has confirmed that after removal of persistent injury factors, the degree of liver fibrosis can be significantly improved. Therefore, comprehensive investigation of the reversibility of HSCs activation is of significant clinical value.^3^ Hepatocytes are the major parenchymal cells of the liver and the impact of stress changes after liver injury on HSCs cannot be overlooked. Further elucidation of the molecular mechanisms underlying HSCs activation after liver injury should facilitate the development of novel strategies for clinical treatment of liver fibrosis.

To date, two hypotheses have been proposed regarding the potential pathways of activation of HSCs, designated ‘two stages’ and ‘three‐step cascade reaction’. The common feature of both theories is that after damage, liver cells secrete cytokines and small vesicles containing complex RNA and proteins that act on HSCs, initiating a series of biochemical reactions within cells. Subsequently, HSCs interact with Kupffer and vascular endothelial cells in the liver, leading to their further transformation into MFs.^4^, ^5^ Hepatocytes account for 60% of the liver volume and are the initial and most important ‘bearers’ of various factors causing chronic injuries, such as viruses and oxidative stress. Therefore, the stress response of liver cells to harmful factors is the initiating step in the activation process of HSCs. Inflammatory molecules released in response to liver cell pyroptosis can trigger activation of HSCs and promote liver fibrosis. Moreover, abnormal expression of signalling proteins in liver cells is closely related to HSCs activation during liver fibrosis.^6^ Activation of hepatocyte‐specific nuclear factor erythroid 2‐related factor 2 (NRF2) is reported to prevent liver fibrosis caused by steatohepatitis.^7^ Moreover, the regulation of specific molecules, including fructose‐1, 6‐bisphosphatase 1 (FBP1), high mobility group protein 1 (HMGB1), and platelet‐derived growth factor receptor (PDGFR), in liver cells can directly alter their expression in HSCs.^8^, ^9^ Therefore, elucidation of the pathways and specific regulatory signals underlying HSC activation by damaged liver cells may provide effective therapeutic targets for treatment of liver fibrosis. In‐depth studies on the mechanisms underlying intercellular information exchange over the years have resulted in an increasing interest in the functions of exosomes from a wide range of sources with strong stability.

Exosomes are small membrane vesicles with a diameter of 40–100 nm secreted by various cells in the body that participate in intercellular communication. Upon release into the microenvironment, exosomes are internalized by target cells and play a role in altering their genetic phenotype. Exosomes from different sources are reported to combat acute and chronic liver injury by improving oxidative stress, reducing the inflammatory response, promoting cell proliferation, and inhibiting apoptosis.^10^ Earlier research has confirmed that lipid damage triggers the release of extracellular vesicles from liver cells, further activating the inflammatory phenotype of macrophages.^11^ After exposure to inflammation stimuli, liver cells secrete extracellular vesicles to mediate toll‐like receptor 3 (TLR3) activation in HSCs, in turn, accelerating liver fibrosis.^12^ In vivo, extracellular vesicles in healthy human serum also contain various microRNAs that inhibit the activation of HSCs, thus exerting significant anti‐liver fibrosis effects.^13^ Long noncoding RNAs (lncRNAs) are noncoding RNAs greater than 200 nucleotides in length, that play important roles in many cellular activities, such as regulation of epigenetic, cell cycle and differentiation processes. In contrast to microRNAs, lncRNAs are usually expressed at low levels in healthy cells and exhibit significant tissue‐and cell‐type specificity during differentiation and development, which is beneficial for the precise regulation of molecular pathways in response to organ damage. Earlier studies indicate that lncRNAs serve as a key factor in the formation and development of human liver fibrosis.^14^ In subsequent reports, multiple lncRNAs have been shown to play key roles in HSC activation and progression of liver fibrosis.^15^ For instance, lncRNA liver fibrosis associated RNA 1 (LFAR1) activates transforming growth factor‐β (TGF‐β) and Notch signalling pathways through phosphorylation of Smad2/3, promoting the occurrence of liver fibrosis.^16^ Extracellular vesicles derived from bile duct cells are rich in lncRNA H19, which is transported to HSCs to alter their phenotype in vitro and in vivo, further supporting key regulatory roles of extracellular vesicle lncRNAs in biological processes.^17^, ^18^

Liver fibrosis is controlled by multiple intracellular signalling pathways. In particular, glial cell line‐derived neurotrophic factor (GDNF) involved in TGF‐β/Smad signalling is critical in regulation of liver fibrosis.^19^, ^20^ Previous studies have identified GDNF as a potential predictive factor and important target for treatment of liver fibrosis.^21^, ^22^ Recent results further showed significant upregulation of GDNF in serum and liver tissues of patients with liver fibrosis, that was positively associated with HSC activation markers, such as α‐smooth muscle actin (α‐SMA), and a regulatory role of GDNF/activin receptor‐like kinase 5 (ALK5)/Smads in HSCs activation.^23^ Due to their significant tissue specificity, lncRNAs may serve as important regulatory factors in the above signalling pathways. This study was performed on CCl_4_‐induced damaged liver cells and mouse liver fibrosis models with specific focus on regulation of the GDNF pathway by lncRNA CYTOR. The main objective was to establish the molecular mechanisms by which damaged liver cells regulate HSC activation through extracellular vesicles, with a view to providing experimental evidence on the regulatory pathways of liver fibrosis in the microenvironment for the development of therapeutic strategies.

## 2 MATERIALS AND METHODS

### 2.1 Cell culture and establishment of a cocultivation model

Normal human liver LO2 and hepatic stellate LX2 cells obtained from the Chinese Academy of Sciences Committee Type Culture Collection cell bank were incubated in Dulbecco's Modified Eagle's Medium (DMEM; Thermo Fisher, USA) containing 10% foetal bovine serum and 100 U/mL streptomycin and penicillin at 37°C and 5% CO_2_. LX2 cells were inoculated into transwell inserts and LO2 cells into cell culture plates for 24 h each. Following removal of culture medium from the transwell chamber and plate, new medium was added to the lower chamber. The transwell chamber was placed in a cell culture plate and new culture medium added. The experimental protocol was performed according to the Declaration of Helsinki and approved by the Ethics Committee of Shidong Hospital.

### 2.2 Isolation of exosomes and cell treatments

Frozen cell culture supernatant was completely thawed in a 25°C water bath and placed on ice for experimental use. Cells were centrifuged at 3000*g* and 10,000*g* at 4°C for 10 min and the supernatant transferred to a new centrifuge tube. After centrifugation at 4°C for 90 min at 100,000*g*, the supernatant was discarded and 30 mL of precooled PBS added for resuspension of the precipitate. Finally, the sample was centrifuged at 100,000*g* and 4°C for 90 min. The supernatant fraction from this step was discarded and PBS (~200 μL) used for resuspension of precipitated exosomes.^24^, ^25^

LO2 cells were treated with CCl_4_ (8 mM) for 24 h to establish a damaged LO2 model (C‐LO2).^26^, ^27^ GW4869 (Sigma Aldrich, USA) was added to cells for 48 h before the collection of exosomes to inhibit exosome production. All exosomes were collected at 72 h after switching to serum‐free culture medium and incubated with the culture medium of target cells at a concentration of 50 μg/mL for 24 h.

### 2.3 Identification and cellular uptake of exosomes

Exosomes were fixed overnight at 4°C with 2.5% glutaraldehyde solution and incubated at room temperature for 5 min, followed by filtration of liquid (Thermo Fisher, USA). The solution was drip‐stained (saturated uranyl acetate solution) onto a copper mesh for 1 min and the liquid filtered. Next, ddH_2_O was dripped onto a copper mesh and allowed to stand at room temperature for 5 min, following which the liquid was filtered with paper. The solution was dried at room temperature and images obtained using transmission electron microscopy (TEM) (JEM‐2100, Japan). A nanoparticle tracking analyser (Zetasizer Nano ZS, UK) was used for particle size detection.

PKH67 linker storage solution (Sigma Aldrich) was diluted 10 times with diluent C to prepare 100 μM dye working solution. To 50 μL extracellular vesicles, a 50 μL aliquot of dye working solution was added and thoroughly mixed. The solution was incubated at room temperature in the dark for 10 min. Exosomes were reextracted with the addition of 10 mL PBS and the sediment resuspended in 100 μL PBS to generate labelled exosomes. Cells were cultured in 96‐well plates (5000 cells per well). After 24 h, labelled extracellular vesicles and cell culture medium were mixed 1:1 ratio into each well and culturing continued for 24 h, using the well without extracellular vesicles as the control. After 24 h of cellular uptake, the culture medium was discarded and 2% paraformaldehyde added to fix cells for 10 min. Cells were washed with PBS three times and incubated with DAPI for 20 min, followed by three further washes with PBS. Images were obtained at 400× magnification under a fluorescence microscope (Olympus, Tokyo, Japan).

### 2.4 RNA sequencing

Total RNA of LO2 and extracellular vesicles of damaged LO2 was extracted, fragmented and reverse‐transcribed for whole genome sequencing. From the perspective of bioinformatics, coexpression analysis based on expression correlation was employed to predict trans target genes regulated by lncRNAs. The Pearson correlation coefficient could be effectively used to predict the relationships between differentially expressed lncRNAs and all coding‐protein mRNAs (satisfying *R* ^2^ ≥ 0.95 and expressed as reading per million mapped reads [RPM]). Expression levels of each lncRNA were compared with the aid of DEGseq and images generated using Sangerbox.^28^

### 2.5 Polymerase chain reaction

Total RNA of treated cells was extracted with TRIzol reagent (Invitrogen, USA) and quantified using the quantitative fluorescence polymerase chain reaction (PCR) reaction system (Takara, China). The PCR operating parameters were set as follows: 95°C for 3 min, 92°C denaturation for 30 s and 60°C annealing for 30 s for a total of 35 cycles. PCR results were obtained using the 2^−ΔΔCt^ method. The nucleotide sequences of the primers used for qRT‐PCR are shown in Table 1.

**TABLE 1:** Nucleotide sequences of primers for qRT‐PCR.

Gene		Primer sequence (5′–3′)
*GDNF*	Forward	GGCAGTGCTTCCTAGAAGAGA
	Reverse	AAGACACAACCCCGGTTTTTG
*miR‐125*	Forward	TCCCTGAGACCCTAACTTGTGA
	Reverse	AGTCTCAGGGTCCGAGGTATTC
*CYTOR*	Forward	CATCCACATTCCAACCTCCGTCTG
	Reverse	TCGGCGGGCAACAGGTAGAG
*U6*	Forward	AAAGCAAATCATCGGACGACC
	Reverse	GTACAACACATTGTTTCCTCGGA
*GAPDH*	Forward	GGAGCGAGATCCCTCCAAAAT
	Reverse	GGCTGTTGTCATACTTCTCATGG

### 2.6 SiRNA transfection and luciferase reporter assay

CYTOR siRNA, miR‐125 mimetics, and miR‐125 inhibitors (RiboBio, Guangzhou, China) were stored as freeze‐dried powder at −20°C and diluted to a stock concentration of 20 μm with sterile RNase‐free H_2_O. LO2 cells were transfected with specific RNA oligonucleotides using Lipofectamine 3000 (Invitrogen, Carlsbad, CA, USA). After 6 h, the medium was replaced with new culture medium.

Cells in each well were transfected with pmir‐GLO‐CYTOR‐3′ UTR (200 ng), pmir‐GLO‐GDNF‐3′ UTR miRNA, mimic (500 ng) and negative carrier (200 ng). Lipofectamine 3000 was diluted with Opti‐MEM medium and incubated at room temperature for 5 min. Next, the transfection reagent was mixed with carrier DNA and mimic, incubated at room temperature for 20 min, and thoroughly mixed with Opti‐MEM medium. Cells were collected after 48 h of cultivation in a 5% CO_2_ incubator at 37°C. Detection was performed using the dual luciferase reporter gene kit according to the manufacturer's instructions (Sigma Aldrich) and the fluorescence value of the carrier itself used as the internal control with a dual‐luciferase reporter system (Promega, Madison, WI, USA). The siRNA nucleotide sequences are shown in Table 2.

**TABLE 2:** Nucleotide sequences of siRNAs specific for CYTOR.

Si‐RNA		Sequence (5′–3′)
*Si‐CYTOR #1*	Forward	GGAAUGGAGGGAAAAUAAAUGATT
	Reverse	UCAUUUAUUUCCCUCCAUUCCTT
*Si‐CYTOR #2*	Forward	GCCUGUCUUCAGAUCUUCACATT
	Reverse	UGUGAAGAUCUGAAGACAGGCTT
*Si‐CYTOR #3*	Forward	GAGAUGAAACAGGAAGCUCUATT
	Reverse	UAGAGCUUCCUGUUUCAUCUCTT

### 2.7 Western blot

Proteins were extracted using radioimmunoprecipitation assay (RIPA) Buffer (Millipore, USA) and concentrations determined via western blot (WB). A 30 μg aliquot of protein was mixed with 5× Mix SDS‐PAGE loading buffer and heated at 95°C for 5 min for denaturation. Electrophoresis was performed using a 12% acrylamide separation gel using the above heated sample (70 V/30 min, 120 V/60 min). After 90 min of constant current wet rotation at 250 mA, the sample was sealed with 5% skimmed milk powder at room temperature for 1 h. The primary antibody was diluted with 5% skimmed milk powder and incubated with the sample overnight at 4°C. Next, the membrane was washed 3 times with TBST for 5 min each time and incubated with fluorescence‐conjugated secondary antibody (DyLight 800, Odyssey, USA) (1:5000) at room temperature for 1 h. The Odyssey two‐colour infrared laser imaging system (LI‐COR Biosciences, USA) was used to scan the membrane for assessment of staining. The following antibodies were used: α‐SMA (ab7817, Abcam, UK), TGF‐β (YT4632, IMMUN, China), Collagen Type I (CoI‐I) (14695‐1‐AP, Proteintech, China), GAPDH (60004‐1‐Ig, Proteintech), CD63 (67605‐1‐Ig, Proteintech) and TSG101 (67381‐1‐Ig, Proteintech).

### 2.8 Fluorescence in situ hybridization and immunofluorescence

Cells were fixed onto specialized slides, placed in drilling solution at room temperature for 10 min and rinsed with PBS. Next, drops of composite digestion working solution (RiboBio, Guangzhou, China) were used to cover the cell surface at room temperature for 10–30 min. After rinsing with PBS, cells were covered with pre‐hybridization working solution and incubated with a dedicated cover glass for 4–8 h at 42°C. Samples were washed in PBS and hybrid working solution added dropwise again with incubation at 42°C for 8–12 h. Anti‐quenching sealing liquid was used to seal the film under a microscope. The excitation wavelength range was 488–492 nm.

Cells were permeabilized with Triton X‐100 (0.5%) at room temperature for 15 min, following which 5% normal serum was dripped onto a glass slide and sealed at room temperature for 1 h. Each slide was treated with a sufficient amount of diluted primary antibody and placed in a wet box with incubation at 4°C overnight. The climbing tablets were soaked with PBST three times for 3 min each time. After absorption of excess liquid on the climbing tablets with absorbent paper, diluted fluorescent secondary antibody was added dropwise, followed by incubation in a wet box at 37°C for 1 h. The climbing tablets were soaked with PBST three times for 3 min each time. After dropwise addition of DAPI, samples were incubated in the dark for 5 min for nuclear staining. The images were obtained under a fluorescence microscope (Olympus, Tokyo, Japan).

### 2.9 Animal experiments

Male C57 mice (20–22 g) were purchased from Shanghai Laboratory Animal Co., Ltd. (SLAC, Shanghai, China). All experimental procedures were approved by the Institutional Animal Use Committee of Shidong Hospital. Mice were intraperitoneally injected with CCl_4_ (1:10 v/v, 1 mL/kg) diluted in olive oil twice a week for 8 weeks (Sigma‐Aldrich).^29^ Exosomes were injected through the tail vein of the mouse model at a dose of 100 μg twice a week for 4 weeks and serum and liver tissue samples obtained for subsequent experiments.^30^

CCl_4_‐induced mice were randomly divided into six groups as follows (*n* = 8): NC + NC‐exo: Normal mice + Normal LO2‐exosomes, NC + C‐exo: Normal mice + CCl_4_‐LO2‐exosomes; NC + si‐exo: Normal mice + si‐CYTOR‐CCl_4_‐LO2‐exosomes; C + NC‐exo: Fibrosis model mice + Normal LO2‐exosomes; C + C‐exo: Fibrosis model mice + CCl_4_‐LO2‐exosomes; C + si‐exo: Fibrosis model mice + si‐CYTOR‐CCl_4_‐LO2‐exosomes.

### 2.10 Haematoxylin and eosin, Masson and Sirius red staining

Fresh liver tissue was embedded in paraffin, sliced and incubated in xylene I for 10 min, xylene II for 10 min, anhydrous ethanol I for 5 min, anhydrous ethanol II for 5 min, 95% alcohol for 5 min, 90% alcohol for 5 min, 80% alcohol for 5 min, 70% alcohol for 5 min and finally washed in distilled water.

After treatment with haematoxylin dye solution for 3–8 min, samples were briefly differentiated with 1% hydrochloric acid alcohol for a few seconds and rinsed with tap water. Slices were stained with eosin solution for 1–3 min and sequentially incubated in 95% alcohol I for 5 min, 95% alcohol II for 5 min, anhydrous ethanol I for 5 min, anhydrous ethanol II for 5 min, xylene I for 5 min, xylene II for 5 min, dehydrated and made transparent. Next, the slices were removed from xylene, slightly dried and sealed with neutral gum. Masson staining was performed using 0.5% iodine alcohol for 10 min, followed by 5% sodium thiosulfate for 5 min. Next, samples were rinsed with running water for 10 min and stained with Weigert's iron haematoxylin for 5–10 min. After differentiation with 1% hydrochloric acid alcohol, samples were stained with acidic fuchsin solution for 5–10 min and treated with 1% phosphomolybdic acid aqueous solution for about 5 min and 1% glacial acetic acid for 1 min, followed by dehydration with 95% alcohol multiple times. After dehydration with anhydrous alcohol, xylene became transparent and neutral gum was used for sealing. For staining with Sirius red, staining solution was initially added to the slices with incubation at room temperature for 1 h, followed by rinsing with running water for 10 s. Next, samples were treated with 0.5% acetic acid for 20 s and dehydrated with anhydrous alcohol. Xylene was used to induce transparency, followed by sealing with neutral gum.

### 2.11 Serum component and immunohistochemical analyses

Alanine aminotransferase (ALT), aspartate aminotransferase (AST), hyaluronic acid (HA) and laminin (LN) contents were determined using an automated chemistry analyser (Olympus AU1000, Japan). Paraffin slices were incubated in a 70°C constant temperature oven and dewaxing and gradient alcohol treatment performed after 90 min. Samples were washed in pure water and transferred to 3% H_2_O_2_ for 10 min. Subsequently, slices were placed in a pressure cooker filled with 0.01 M citric acid buffer, subjected to high‐pressure repair for 2.5 min, and transferred to cold water for cooling. The slices were incubated in prepared serum of the same origin as the first antibody and sealed at room temperature for 15–30 min. Diluted primary antibody was incubated with tissue slices overnight at 4°C. Biotin‐labelled secondary antibodies were added dropwise and incubated at room temperature or 37°C for 30 min to 1 h, followed by rinsing with PBS. Images were obtained after 5–10 min of diaminobenzidine (DAB) staining.

### 2.12 Statistical analysis

All data are expressed as mean ± standard deviation (SD). Statistical analysis was performed using SPSS 20.0 software (Chicago, IL, USA). One‐way analysis of variance (ANOVA) was used to compare differences among three groups and Student–Newman–Keuls (SNK) test used for intergroup comparisons. *p*‐values <0.05 were considered statistically significant.

## 3 RESULTS

### 3.1 Establishment of a coculture model of LO2 and LX2 and identification of exosomes

**FIGURE 1:**
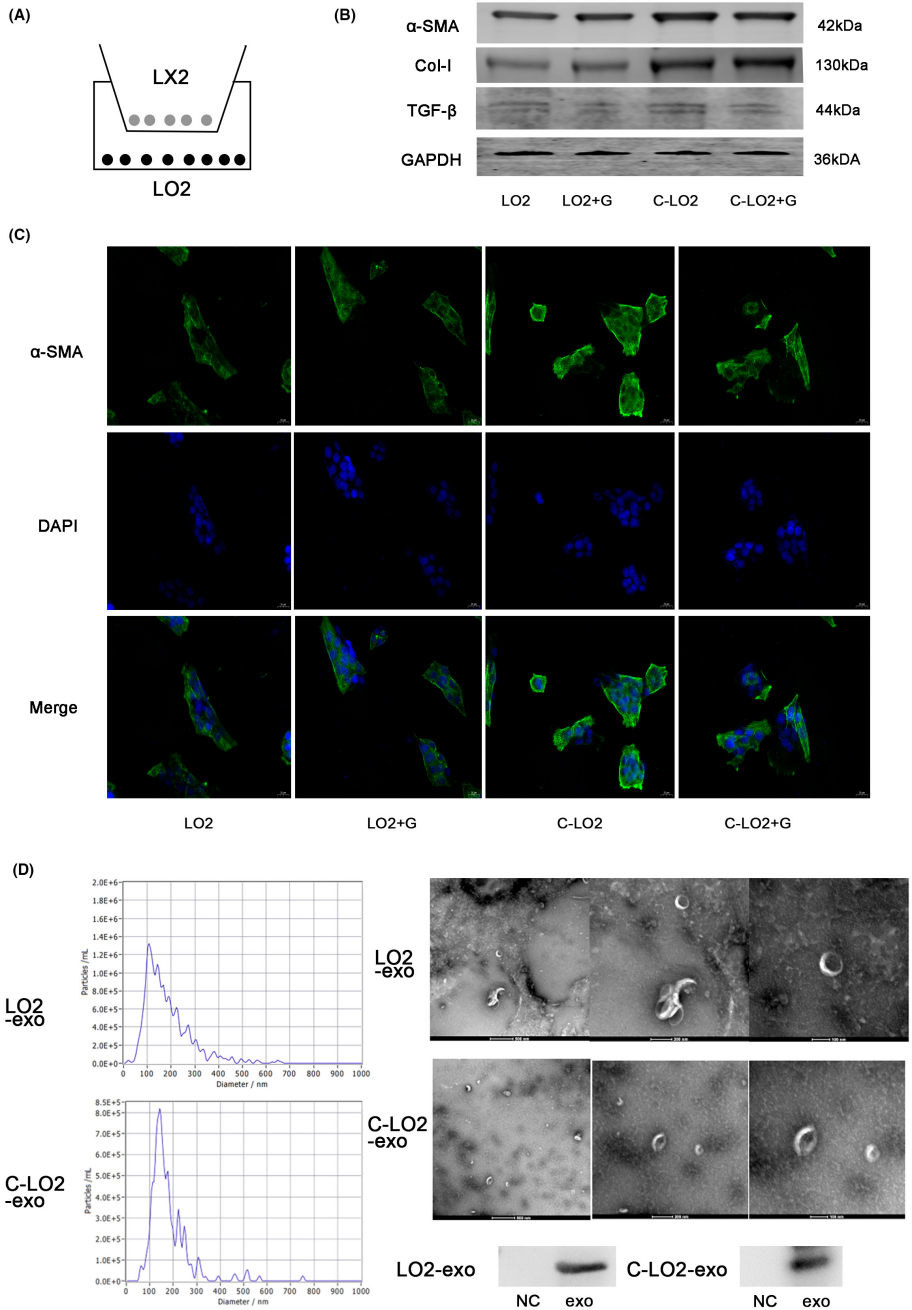
Establishment of a co‐culture model of LO2 and LX2 and identification of exosomes. (A) Schematic diagram of the LO2 and LX2 coculture model. (B) Assessment of protein levels of α‐SMA, CoI‐1 and TGF‐β via western blot (WB). (C) Immunofluorescence staining of α‐SMA expression in LX2 cells (400×). (D) Exosomes were identified via transmission electron microscopy (TEM; 30,000×) and nanoparticle tracking analysis (NTA). Protein levels of CD63 were measured via western blot (WB). *C*‐*LO2*: *CCl*_4_ *‐LO2*; G: *GW4869*.

We established a coculture model of LX2 and LO2 (Figure 1A) and simulated the mechanism of liver fibrosis formation in vivo using CCl_4_ to generate a damaged LO2 model. Expression of liver fibrosis‐related markers (α‐SMA, CoI‐1 and TGF‐β) was increased in LX2 cocultured with damaged LO2, which decreased following addition of the exosome inhibitor GW4869 (Figure 1B). Immunofluorescence results revealed a similar trend of α‐SMA expression (Figure 1C). Exosomes were further isolated for characterization. Notably, the diameter of normal LO2 exosomes was ~110 nm while that of damaged LO2 exosomes was ~140 nm. The exosomes had a clear membrane like or cup‐shaped structure with distinct edges and uniform distribution without aggregation. WB results showed positive expression of the extracellular surface marker CD63 (Figure 1D).

### 3.2 Screening of differentially expressed lncRNAs in exosomes

**FIGURE 2:**
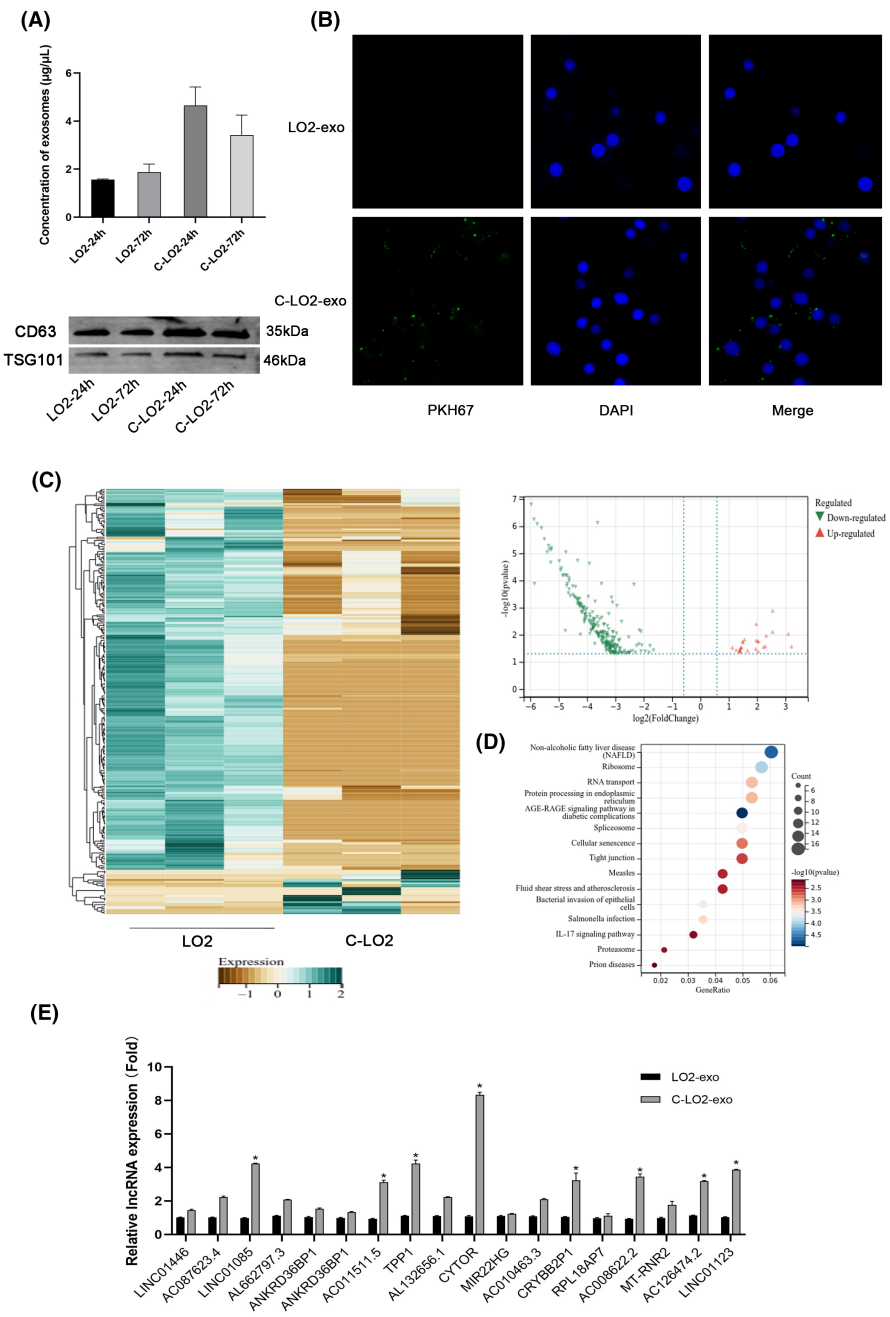
Screening of differential long noncoding RNAs (lncRNAs) in exosomes. (A) BCA analysis of exosome concentrations. Protein levels of CD63 and TSG101 were measured via western blot (WB). (B) Detection of exosome uptake under a fluorescence microscope (400×). (C) Volcano plot and clustered heatmap comparing lncRNA expression patterns. (D) KEGG analysis of DEGs. LncRNAs were classified according to Pearson correlation. (E) qRT‐PCR analysis of relative expression levels of lncRNAs in exosomes. *p < 0.05 for C‐LO2‐exo versus LO2‐exo. C‐*LO2*: *CCl*_4_ *‐LO2*.

We collected and quantified extracellular vesicles at 24 and 72 h after LO2 stimulation. A greater number of exosomes from damaged cells (C‐LO2) and sufficient extracellular vesicles were collected at 24 h (Figure 2A). Exosomes were further stained with PKH26 and added to LX2. After co‐culturing for 24 h, exosomes clustered around the cells and were absorbed (Figure 2B). Extraction of RNA sequences from exosomes of LO2 and C‐LO2 cells revealed significant differences in lncRNAs between healthy and damaged cells. Overall, we identified 246 differentially expressed genes based on log2 (multiple change)>1 (*p* < 0.05, and FDR <0.05), among which 21 were upregulated and 235 were downregulated.^28^ Kyoto Encyclopedia of Genes and Genomes (KEGG) pathway analysis was conducted on differentially expressed genes and a total of 18 lncRNAs related to liver steatosis screened (Figure 2D). Further examination of lncRNA expression in exosomes revealed the most significant difference in lncRNA CYTOR (Figure 2E). Based on these results, we infer that exosomes transfer the lncRNA CYTOR to LX2 for initiation of activation.

### 3.3 Validation and functional regulation of CYTOR

**FIGURE 3:**
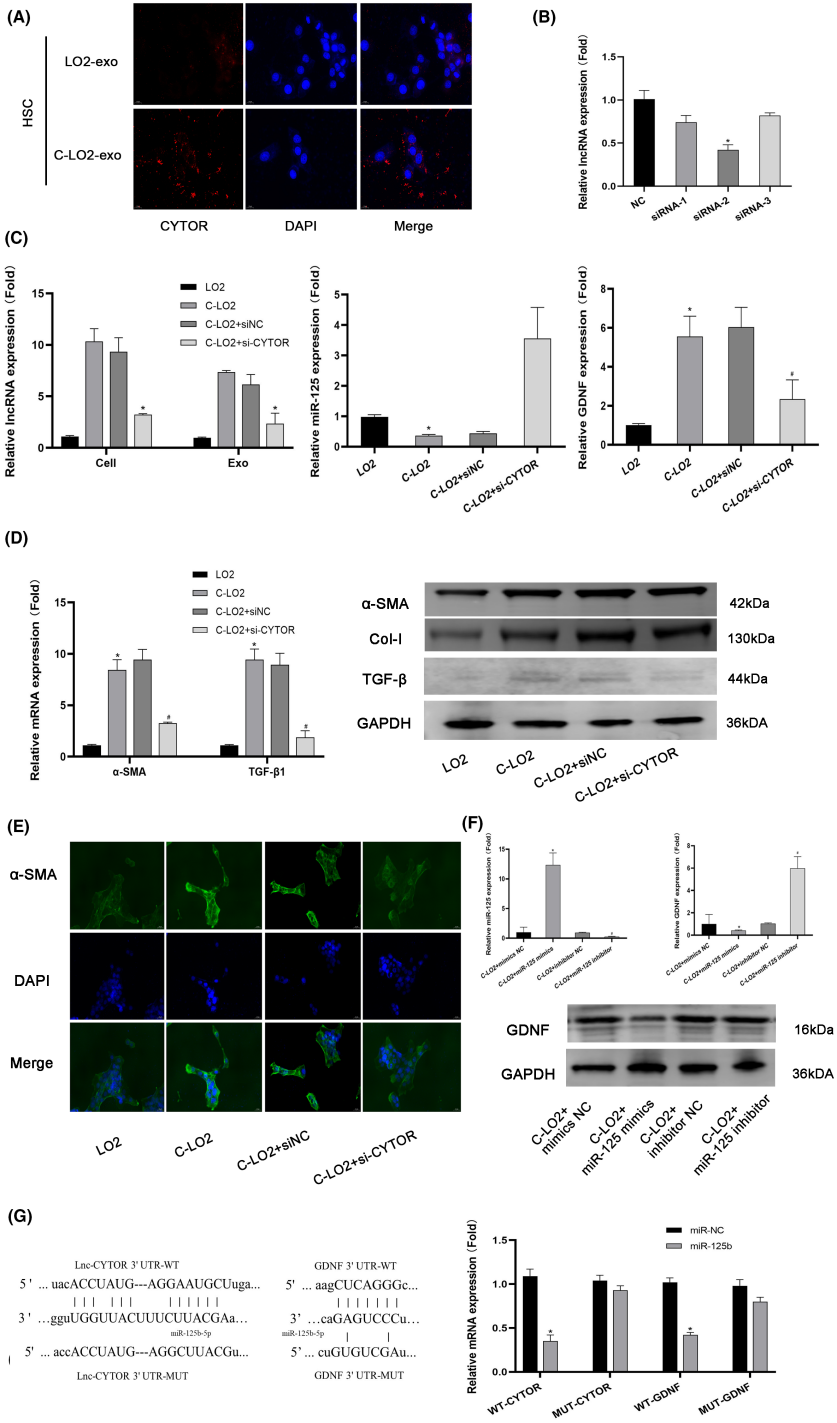
Verification and functional regulation of CYTOR. (A) The location of CYTOR established using FISH (400×). (B) qRT‐PCR assessment of relative expression levels of CYTOR. *p < 0.05 for siRNA‐2 versus NC. (C) qRT‐PCR assessment of relative expression levels of CYTOR, miR‐125 and GDNF. *^#^ *p* < 0.05 for C‐LO2 + si‐CYTOR versus C‐LO2 + siNC. (D) qRT‐PCR analysis of relative expression levels of α‐SMA and TGF‐β. Protein levels of α‐SMA, CoI‐1 and TGF‐β were measured via western blot (WB). *p < 0.05 for C‐LO2 versus LO2. ^#^ *p* < 0.05 for C‐LO2 + si‐CYTOR versus C‐LO2 + siNC. (E) Expression of α‐SMA in LX2 cells detected via immunofluorescence staining (400×). (F) qRT‐PCR assessment of relative expression levels of miR‐125 and GDNF. Protein levels of GDNF were measured via western blot (WB). *p < 0.05 for C‐LO2 + miR‐125 mimic versus C‐LO2 + mimic NC. ^#^ *p* < 0.05 for C‐LO2 + miR‐125 inhibitor versus C‐LO2 + inhibitor NC. (G) Sequences of wild‐type and mutant CYTOR and GDNF that disrupt interactions with miR‐125 are shown. Relative dual luciferase activity is presented. *p < 0.05 for miR‐125 versus miR‐NC. *C*‐*LO2*: *CCl*_4_‐*LO2*.

Fluorescence in situ hybridization (FISH) experiments revealed that CYTOR is located in the cytoplasm of LX2 cells stimulated by exosomes secreted by damaged liver cells (Figure 3A). Next, we synthesized siRNA specific for CYTOR for validation experiments (Figure 3B). Specifically, siRNA was added to C‐LO2 and the exosomes derived were extracted and added to the corresponding LX2 cells. Upon siRNA‐mediated regulation, CYTOR synchronization between cells and exosomes decreased. Correspondingly, the miR‐125 level increased while expression of GDNF was decreased (Figure 2C). To explore the changes in LX2 function, we further examined the gene and protein expression of liver fibrosis‐related biomarkers. Upon downregulation of CYTOR in damaged liver cells, expression levels of liver fibrosis‐related biomarkers were decreased. Fluorescence expression patterns of α‐SMA confirmed the above findings (Figure 3E). To further validate the downstream pathways, mimics and inhibitors of miR‐125 were employed, which revealed synchronous expression of GDNF. These findings were verified via the luciferase assay showing that CYTOR and miR‐125 as well as miR‐125 and GDNF target and regulate each other.

### 3.4 Role and mechanism of action of extracellular vesicles in mice with liver fibrosis

**FIGURE 4:**
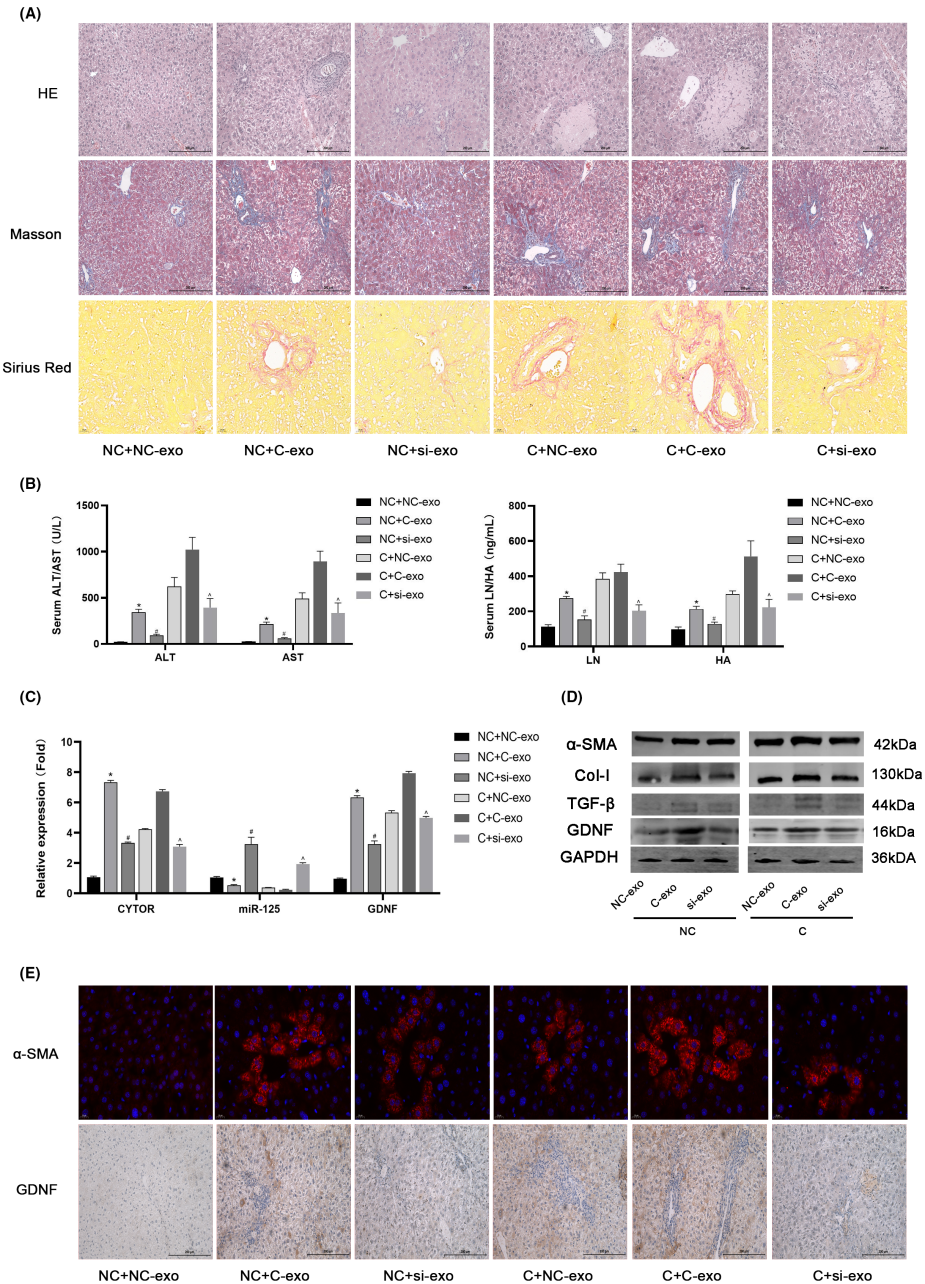
Role and mechanism of action of extracellular vesicles in liver fibrosis in mice. (A) Representative HE‐, Masson‐and Sirius red‐stained liver sections examined via digital microscopy (200×). (B) Serum levels of ALT, AST, LN and HA. *p < 0.05 for NC + C‐exo versus NC + NC‐exo. ^#^ *p* < 0.05 for NC + si‐exo versus NC + C‐exo. ^^^ *p* < 0.05 for C + si‐exo versus C + C‐exo. (C) qRT‐PCR assessment of relative expression levels of CYTOR, miR‐125 and GDNF. *p < 0.05 for NC + C‐exo versus NC + NC‐exo. ^#^ *p* < 0.05 for NC + si‐exo versus NC + C‐exo. ^^^ *p* < 0.05 for C + si‐exo versus C + C‐exo. (D) Western blot analysis of protein levels of α‐SMA, CoI‐1, TGF‐β and GDNF. (E) Immunofluorescence and immunohistochemical staining for evaluation of α‐SMA and GDNF expression (400×). C: *CCl*_4_‐*mice*; *C*‐*exo: CCl4*‐*LO2‐exo*; *si*‐*exo*: *siRNA*‐*LO2*‐*exo*.

First, a mouse model of liver fibrosis was established. NC‐exo and C‐exo were injected into normal and model mice, respectively, with the addition of siRNA‐CYTOR regulatory factors. Upon addition of exosomes from damaged liver cells to normal cells, HE, Masson, and Sirius red staining results indicated mild damage, collagen fibre and liver fibrosis formation. After downregulation of CYTOR, the degree of fibrosis was decreased. Addition of exosomes from normal cells to the model could alleviate liver fibrosis to a certain extent, with enhanced improvement following siRNA treatment (Figure 4A). In keeping with pathological and staining results, liver enzyme parameters (serum ALT and AST) as well as fibrosis‐related indicators (LN and HA) were generally higher than normal in model animals. Exosomes from damaged cells exacerbated liver fibrosis, while downregulation of CYTOR alleviated liver fibrosis (Figure 3B). We validated the gene and protein expression of components of downstream pathways with the aid of qPCR, WB, immunofluorescence and histochemistry (Figure 3C,D). Our results are consistent with the above findings, clearly indicating that downregulation of CYTOR could effectively inhibit the formation of liver fibrosis in which exosomes play an important delivery role.

## 4 DISCUSSION

Significant progress has been made in studies on molecular signalling regulated by HSCs and related targeted drugs that play an important clinical role. However, indiscriminate molecular interventions can seriously affect the growth and development of other tissues and organs in the body.^31^, ^32^ Therefore, clarification of the upstream signals activated by HSCs and screening for liver‐specific regulatory factors is critical for effective treatment of liver fibrosis.

In this study, we constructed a replicable coculture model of LO2 and LX2 and simulated liver cell damage in vivo using CCl_4_. Our results showed that damaged liver cells could induce LX2 activation and upregulate liver fibrosis‐related markers. Accordingly, we speculate that specific components within the supernatant transmit to related signalling pathways. Accumulating evidence suggests that exosomes serve as important connections between cells. Compared with stem cell transplantation, exosomes have stronger stability as well as lower immune rejection and tumorigenicity. In addition, developments in freeze‐drying purification and nanotechnology have provided more favourable conditions for the long‐term storage and targeted delivery of extracellular vesicles. In this study, we extracted and identified exosomes from the supernatant and confirmed through cell uptake experiments that extracellular vesicles enter LX2 cells, which could exert important downstream regulatory effects. Exosomes are rich in ncRNAs and proteins, which mediate the functions of multiple cells through various mechanisms.^33^, ^34^ Long noncoding RNAs (lncRNAs) have become an important direction in the field of precise regulation due to their cell and tissue specificity.^35^ For example, exosomal miR‐192‐5p secreted by bone marrow mesenchymal stem cells inhibits hepatic stellate cell activation and targets protein phosphatase 2‐regulatory subunit 3A (PPP2R3A).^36^ Moreover, hepatocyte‐derived exosomes have been shown to deliver H2A histone family‐member J (H2AFJ) and Ninjurin2 to HSCs and promote liver fibrosis.^37^, ^38^

**FIGURE 5:**
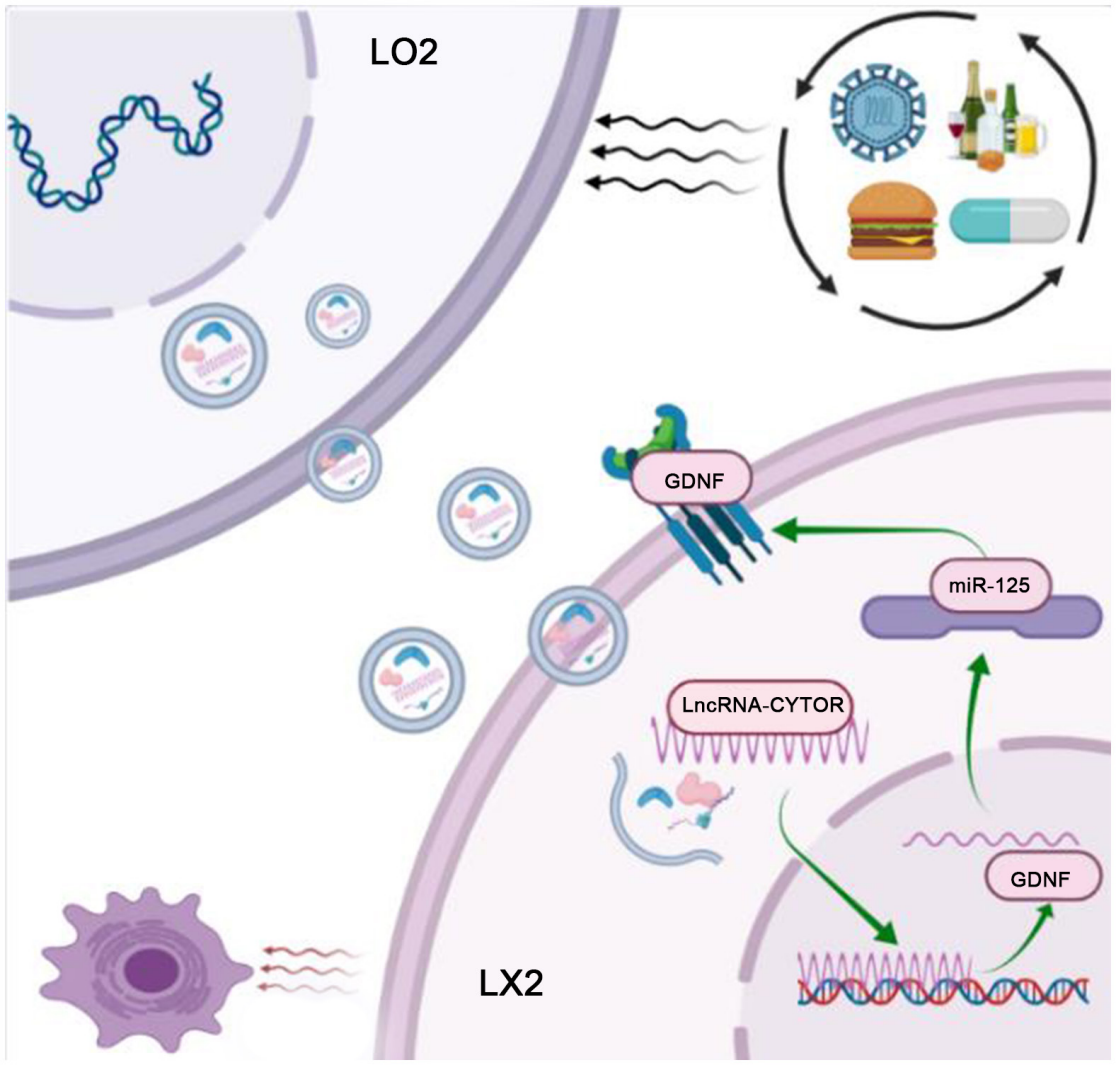
Mechanism of action of CYTOR in exosomes. Upregulated CYTOR is enveloped by exosomes and enters the cytoplasm of LX2, competing with miR‐125 by acting as ceRNA to inhibit GDNF and regulate activation of hepatic stellate cells. The lncRNA CYTOR provides a specific and sensitive regulatory circuit for HSC activation by regulating downstream GDNF expression (By Figdraw.).

We compared the exosomes of damaged and normal liver cells and further conducted gene screening to identify differences in expression of transported genes. The lncRNA CYTOR was identified as the most differentially expressed gene and its localization and expression further validated. Based on the data, we propose that upregulated lncRNA CYTOR is encapsulated by exosomes and translocates into the cytoplasm of LX2, where it plays an important biological role. However, regulation of nuclear expression may potentially occur through a ceRNA‐mediated mechanism. Subsequently, miR‐125b and GDNF were identified as closely related molecules involved in regulation of lncRNA CYTOR, which was validated in subsequent molecular pathway analyses. Upon downregulation of lncRNA CYTOR in cells and exosomes, the corresponding miR‐125b level, increased while GDNF expression was decreased, leading to inhibition of LX2 activation accompanied by a decrease in biomarker levels. As validated using the luciferase assay, lncRNA CYTOR and miR‐125b as well as miR‐125b and GDNF could target and regulate each other. Our data indicate that LO2 transmits lncRNA signals through exosomes, affecting the activation of LX2. With regard to its mechanism of action, lnc CYTOR may act as a ceRNA that competes with miR‐125 to inhibit GDNF and regulate activation of HSCs (Figure 5). Previous studies have demonstrated that lncRNAs can play regulatory roles through by functioning as competing endogenous RNAs (ceRNAs). For instance, Zou and colleagues showed that lncRNAs activated by DNA damage inhibition suppress HSC activation via the microRNA‐495‐3p/sphingosine 1‐phosphate acceptor 3 axis.^39^ Similarly, other studies have demonstrated that lncRNA maternally expressed 3 (MEG3) modulates HSC activation by spreading miR‐145 to regulate peroxisome proliferator activated receptor (PPAR) gamma.^40^

Finally, we validated the mechanism of action of lncRNA CYTOR in liver fibrosis in vivo. Exosomes secreted by normal and damaged liver cells were injected into normal and model mice as well as exosomes with downregulated levels of lncRNA CYTOR. Normal exosomes exerted therapeutic effects on liver fibrosis. Additionally, silencing of lncRNA CYTOR reduced the degree of liver fibrosis. Consistent with in vitro cytological results, our in vivo data showed that levels of miR‐125b and GDNF as well as markers of liver fibrosis underwent changes dependent on the expression of lncRNA CYTOR. Based on the collective findings, we propose that downregulation of lncRNA CYTOR could effectively inhibit the formation of liver fibrosis in vivo, in which exosomes play an important role. Exosomes exhibit excellent biocompatibility and low immunogenicity, thereby effectively mitigating rejection and minimizing adverse effects in patients undergoing treatment. Moreover, exosomes possess the ability to modulate cell signalling through the transportation of bioactive substances, resulting in a favourable therapeutic outcome. Additionally, exosomes can enhance therapy precision and efficacy by facilitating targeted release of drugs or bioactive substances. However, exosomes still have limitations as a therapeutic intervention for diseases. First, the acquisition and purification process of exosomes is intricate and costly, thereby impeding their widespread application. Second, exosomes necessitate stringent stability and storage conditions, rendering them susceptible to environmental factors that compromise their activity. Additionally, further clinical validation and research support are imperative to ascertain the efficacy and safety of exosome therapy.

## 5 CONCLUSIONS

Our results suggest that exosomes of damaged liver cells may act as a ‘transmission pathway’ to deliver endogenous ‘grippers’ (lncRNA CYTOR) to HSCs that regulate the expression of downstream GDNF through ceRNA activity. Elucidation of the molecular pathways by which damaged liver cells regulate HSC activation through extracellular vesicles should facilitate the development of a more specific and sensitive regulatory approach for reversing liver fibrosis.

## AUTHOR CONTRIBUTIONS

**Wenqiang Xu:** Methodology (equal); writing – original draft (lead). **Wenhui Mo:** Data curation (lead); methodology (equal); visualization (lead). **Dengyu Han:** Data curation (equal); software (lead). **Weiqi Dai:** Funding acquisition (equal); supervision (equal); writing – review and editing (equal). **Xiaorong Xu:** Funding acquisition (equal); supervision (equal); writing – review and editing (equal). **Jingjing Li:** Funding acquisition (equal); methodology (equal); writing – review and editing (equal). **Xuanfu Xu:** Supervision (lead); writing – review and editing (lead).

## FUNDING INFORMATION

This study was supported by the National Natural Science Foundation of China (nos 82100638, 81772591, 81970554 and 82002539).

## CONFLICT OF INTEREST STATEMENT

The authors have declared no conflict of interest.

## Data Availability

Data openly available in a public repository that issues datasets with DOIs.
